# Supplementary material for: Distinct clinical and immunological profiles of patients with evidence of SARS-CoV-2 infection in sub-Saharan Africa
Source: Nat Commun. 2021 Jun 11;12:3554. doi: 10.1038/s41467-021-23267-w (PMC8196064; doi:10.1038/s41467-021-23267-w)
Supplement: Supplementary file 1 — Supplementary Information [file 41467_2021_23267_MOESM1_ESM.pdf]

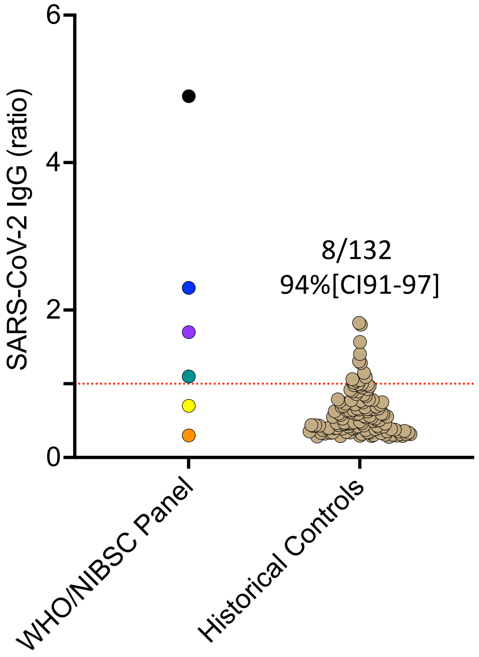

Plasma	NIBSC Reference Result	Omega Diagnostic ELISA Result
**20/130**	Positive	Positive
**20/120**	Positive	Positive
**20/124**	Positive	Positive
**20/122**	Positive	Positive
**20/126**	Negative	Negative
**20/128**	Negative	Negative

Supplementary Figure 1. Verification of SARS-CoV-2 ELISA kit. Pre-pandemic historical samples (2016-2019) and NIBSC reference plasma were tested for SARS-CoV-2 S2 and NP IgG antibodies using the Omega Diagnostic SARS-CoV-2 ELISA kit. The data are reported as the ratio of OD in the test samples to the assay threshold control (Historical samples, n=132; NIBSC reference plasma, n=6). Source data are provided as a Source Data file.

Nasal lining fluid

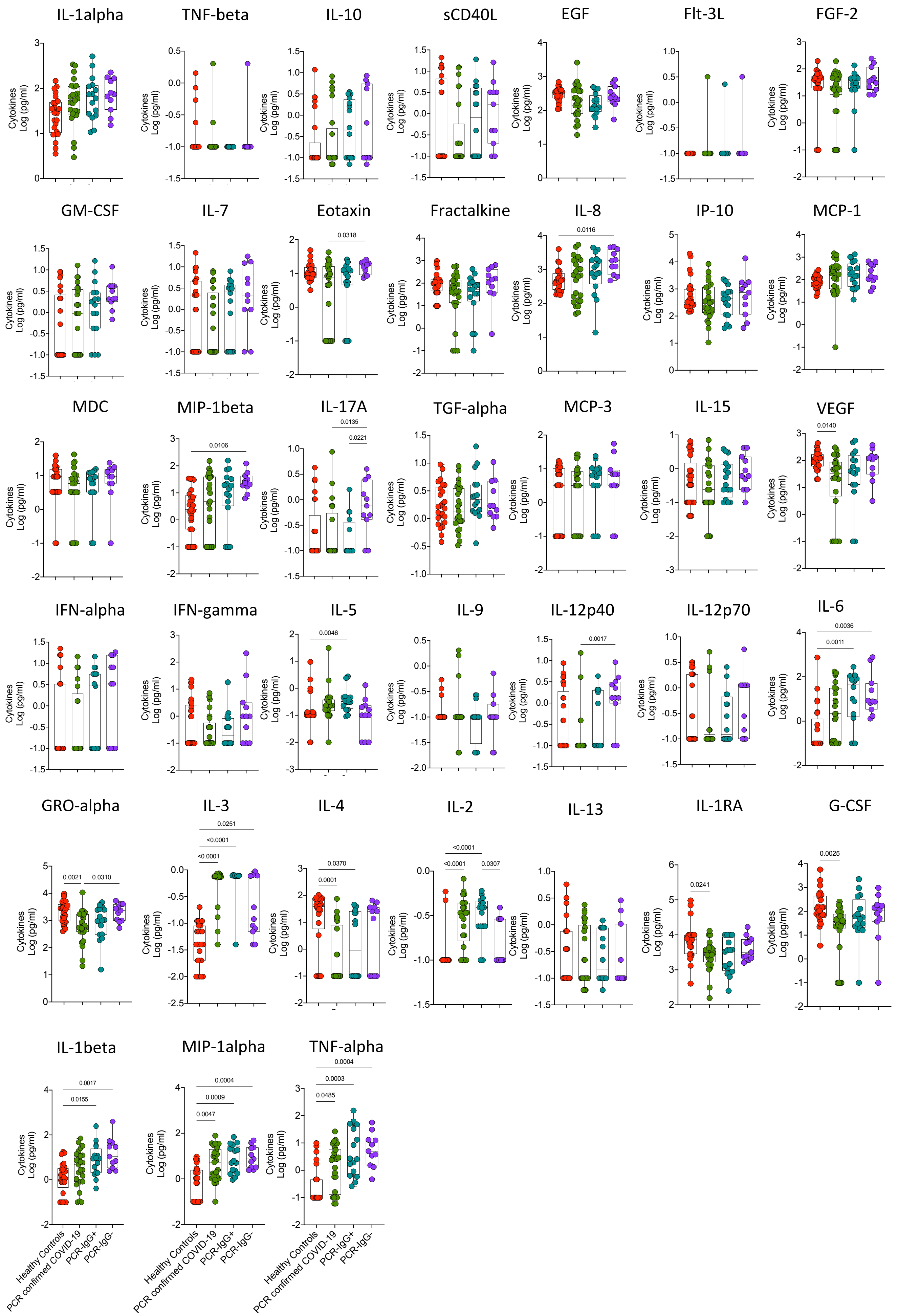

Supplementary Figure 2. Levels of analytes in nasal lining fluid. For all boxplots, box boundaries correspond to 25th and 75th percentiles; whiskers extend to a maximum or minimum greatest value. Data were analysed using Kruskal-Wallis test, two-sided (Healthy controls, n=25; PCR confirmed COVID-19; n=25; PCR-/IgG+ SARI, n=16; PCR-/IgG- SARI, n=11). SARS-CoV-2, severe acute respiratory syndrome coronavirus 2; COVID-19, coronavirus disease of 2019; PCR, polymerase chain reaction; IgG, immunoglobulin G; SARI, severe acute respiratory infection. Source data are provided as a Source Data file.

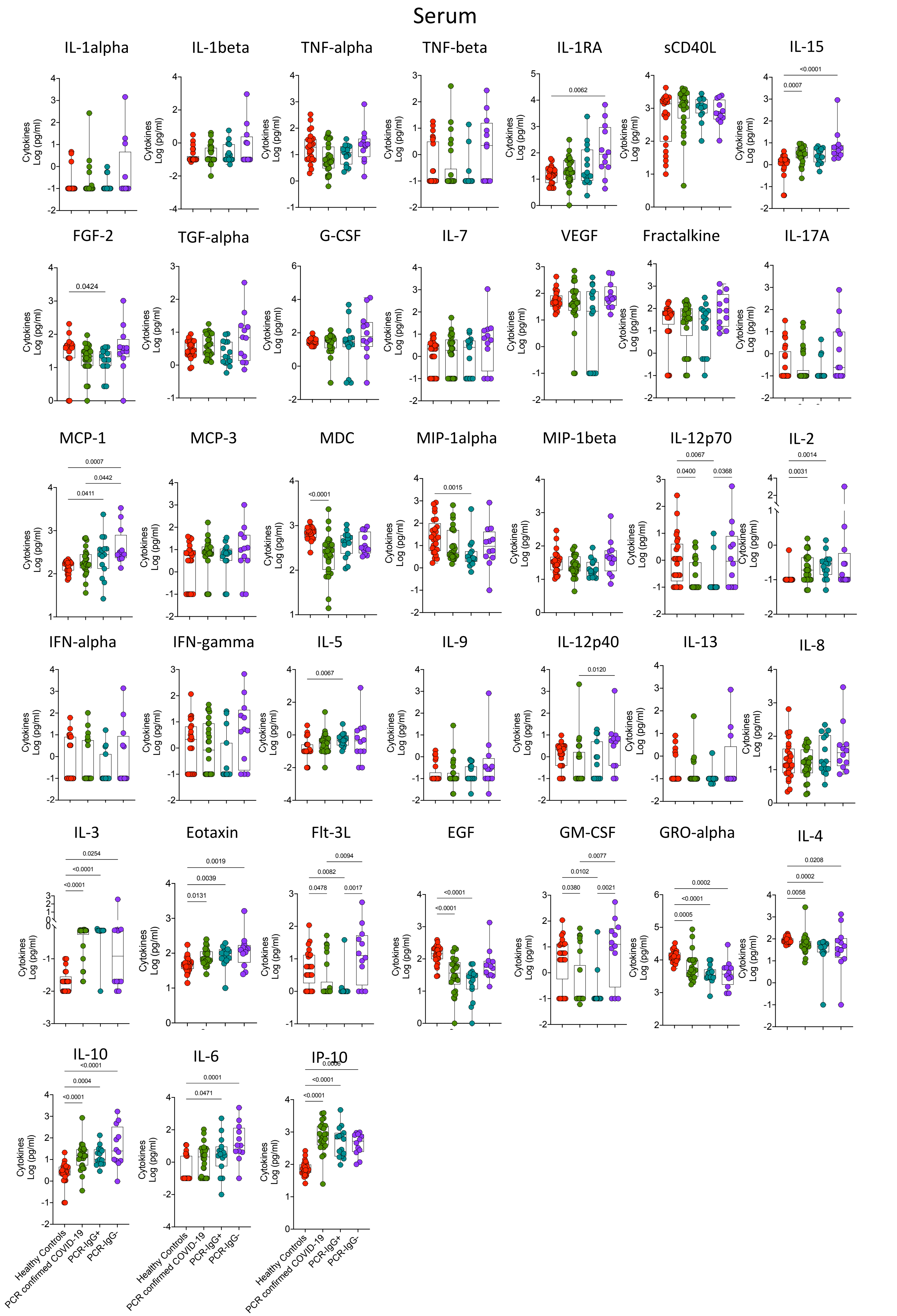

Serum
IL-1alpha
IL-1beta
TNF-alpha
TNF-beta
IL-1RA
0.0062
sCD40L
IL-15
<0.0001
0.0007
FGF-2
0.0424
TGF-alpha
G-CSF
IL-7
VEGF
Fractalkine
IL-17A
MCP-1
0.0007
0.0442
0.0411
MCP-3
MDC
<0.0001
MIP-1alpha
0.0015
MIP-1beta
IL-12p70
0.0067
0.0400
0.0368
IL-2
0.0014
0.0031
IFN-alpha
IFN-gamma
IL-5
0.0067
IL-9
IL-12p40
0.0120
IL-13
IL-8
IL-3
0.0254
<0.0001
<0.0001
Eotaxin
0.0019
0.0039
0.0131
Flt-3L
0.0094
0.0082
0.0478
0.0017
EGF
<0.0001
<0.0001
GM-CSF
0.0077
0.0102
0.0380
0.0021
GRO-alpha
0.0002
<0.0001
0.0005
IL-4
0.0208
0.0002
0.0058
IL-10
<0.0001
0.0004
<0.0001
IL-6
0.0001
0.0471
IP-10
0.0006
<0.0001
<0.0001
Cytokines Log (pg/ml)
Healthy Controls
PCR confirmed COVID-19
PCR-IgG+
PCR-IgG-

Supplementary Figure 3. Levels of analytes in serum. For all boxplots, box boundaries correspond to 25th and 75th percentiles; whiskers extend to a maximum or minimum greatest value. Data were analysed using Kruskal-Wallis test, two-sided (Healthy controls, n=25; PCR confirmed COVID-19; n=25; PCR-/IgG+ SARI, n=16; PCR-/IgG- SARI, n=11). SARS-CoV-2, severe acute respiratory syndrome coronavirus 2; COVID-19, coronavirus disease of 2019; PCR, polymerase chain reaction; IgG, immunoglobulin G; SARI, severe acute respiratory infection. Source data are provided as a Source Data file.

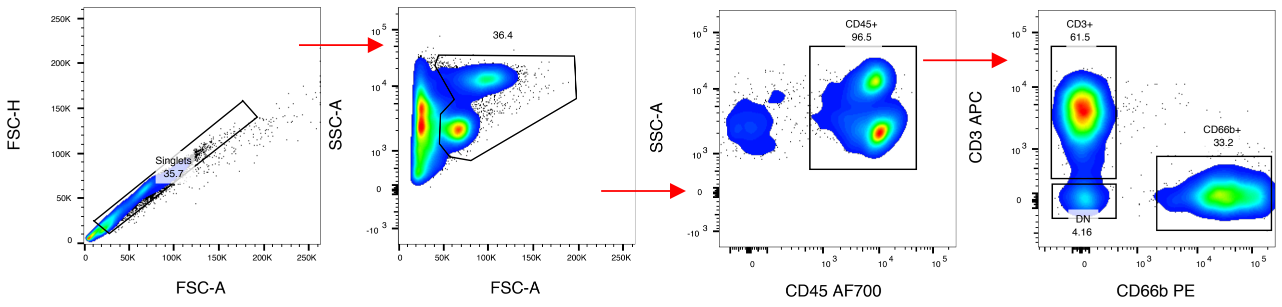

Supplementary Figure 4. Gating strategy for phenotyping nasal immune cells. Gating strategy to determine the percentage of neutrophils (CDD66b+) and T cells (CD3+) in nasal cells.

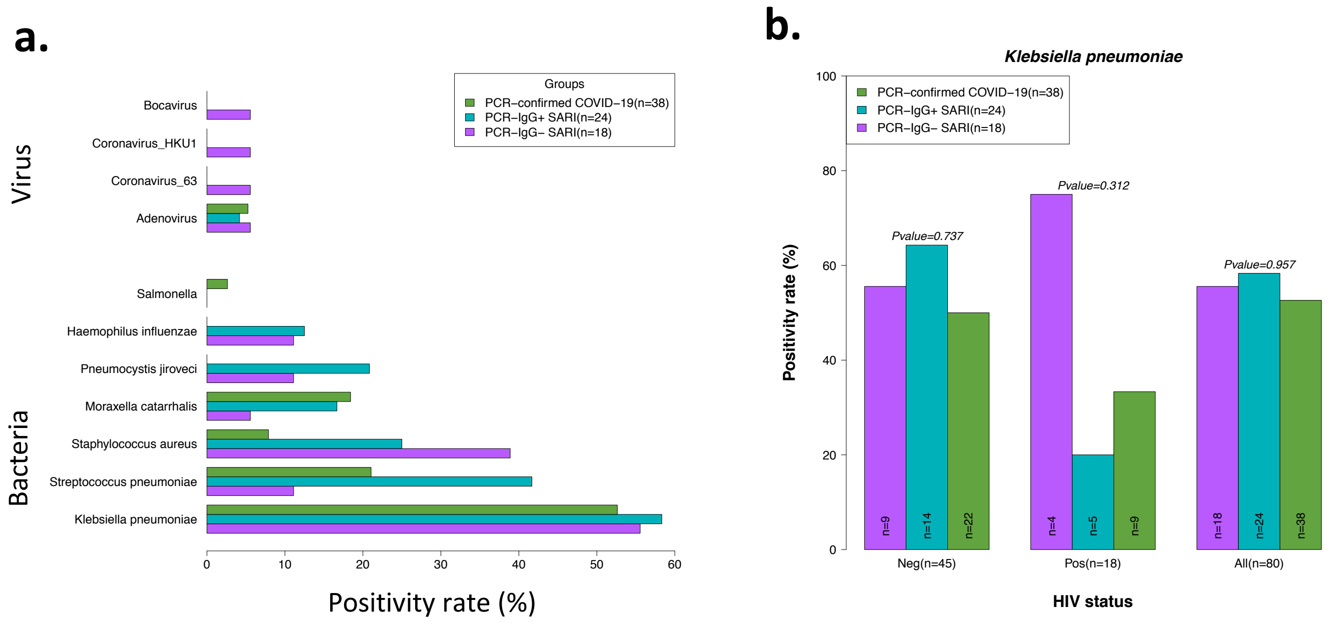

Supplementary Figure 5. Prevalence of co-colonisation/infection in suspected and confirmed COVID-19 based on HIV status. a) Prevalence rate of pathogens per study group. b) Prevalence of Klebsiella pneumoniae co-colonisation in suspected and confirmed COVID-19 in those with and without HIV infection. Data were analysed using Kruskal-Wallis test, two-sided (PCR-confirmed COVID-19, n=38; PCR-/IgG+ SARI, n=24; PCR-/IgG- SARI, n=18). SARS-CoV-2, severe acute respiratory syndrome coronavirus 2; COVID-19, coronavirus disease of 2019; PCR, polymerase chain reaction; IgG, immunoglobulin G; SARI, severe acute respiratory infection. Source data are provided as a Source Data file.
