# Supplementary material for: Distinct clinical and immunological profiles of patients with evidence of SARS-CoV-2 infection in sub-Saharan Africa
Source: Nat Commun. 2021 Jun 11;12:3554. doi: 10.1038/s41467-021-23267-w (PMC8196064; doi:10.1038/s41467-021-23267-w)
Supplement: Supplementary file 3 — Reporting Summary [file 41467_2021_23267_MOESM3_ESM.pdf]

nature research

Corresponding author(s): Ben Morton
Kondwani Jambo

Last updated by author(s): Apr 21, 2021

Reporting Summary

Nature Research wishes to improve the reproducibility of the work that we publish. This form provides structure for consistency and transparency in reporting. For further information on Nature Research policies, see our Editorial Policies and the Editorial Policy Checklist.

Statistics

For all statistical analyses, confirm that the following items are present in the figure legend, table legend, main text, or Methods section.

n/a	Confirmed	
☐	☒	The exact sample size (*n*) for each experimental group/condition, given as a discrete number and unit of measurement
☐	☒	A statement on whether measurements were taken from distinct samples or whether the same sample was measured repeatedly
☐	☒	The statistical test(s) used AND whether they are one- or two-sided<br>*Only common tests should be described solely by name; describe more complex techniques in the Methods section.*
☒	☐	A description of all covariates tested
☐	☒	A description of any assumptions or corrections, such as tests of normality and adjustment for multiple comparisons
☐	☒	A full description of the statistical parameters including central tendency (e.g. means) or other basic estimates (e.g. regression coefficient) AND variation (e.g. standard deviation) or associated estimates of uncertainty (e.g. confidence intervals)
☐	☒	For null hypothesis testing, the test statistic (e.g. *F*, *t*, *r*) with confidence intervals, effect sizes, degrees of freedom and *P* value noted<br>*Give P values as exact values whenever suitable.*
☐	☒	For Bayesian analysis, information on the choice of priors and Markov chain Monte Carlo settings
☒	☐	For hierarchical and complex designs, identification of the appropriate level for tests and full reporting of outcomes
☐	☒	Estimates of effect sizes (e.g. Cohen's *d*, Pearson's *r*), indicating how they were calculated

Our web collection on statistics for biologists contains articles on many of the points above.

Software and code

Policy information about availability of computer code

Data collection	Clinical REDCap v9.5.0; Flow Cytometry BD FACS DIVA v9.0 (BD Biosciences); Luminex xPONENT v4.2 (Luminex); ELISA Gen 5 v2.09 (BioTek); QuantStudio Real-Time PCR Software v1.3 (Applied Biosytems)
Data analysis	GraphPad Prism v9.0.1; Stata v15; FlowJo v10.5.3; R (v4.0.0) using ggplot2 (v3.3.2), ggfortify (v0.4.11) and corrplot (v0.84); RStudio v1.1.447

For manuscripts utilizing custom algorithms or software that are central to the research but not yet described in published literature, software must be made available to editors and reviewers. We strongly encourage code deposition in a community repository (e.g. GitHub). See the Nature Research guidelines for submitting code & software for further information.

Data

Policy information about availability of data

All manuscripts must include a data availability statement. This statement should provide the following information, where applicable:

- Accession codes, unique identifiers, or web links for publicly available datasets
- A list of figures that have associated raw data
- A description of any restrictions on data availability

The authors declare that the data supporting the findings of this study are available within the paper and its supplementary information files. Source data are provided with the paper.

Field-specific reporting

Please select the one below that is the best fit for your research. If you are not sure, read the appropriate sections before making your selection.

- [x] Life sciences
- [ ] Behavioural & social sciences
- [ ] Ecological, evolutionary & environmental sciences

For a reference copy of the document with all sections, see nature.com/documents/nr-reporting-summary-flat.pdf

Life sciences study design

All studies must disclose on these points even when the disclosure is negative.

Sample size	We approached all hospitalized patients that met our severe acute respiratory infection criterion and obtained informed consent from those that were interested to join the study. Recruitment was halted when the cases of patients meeting our criteria had dropped drastically within the hospital and nationally at the end of the first wave of the pandemic in Malawi. Whilst recruitment was opportunistic and no sample size was formally calculated we suggest that the sample is sufficient based on previous studies on respiratory cytokine analysis in a similar study setting (Jambo et al. ERJ Open Res. 2017 Oct; 3(4): 00097-2017.)
Data exclusions	No data was excluded from the analysis
Replication	Individuals samples for the antibody and cytokine assays were run once but in batches. Findings can not be replicated for all flow cytometry experiments as all cells were used. However, for the antibody assays on serum, and cytokine responses in nasal and serum samples, the samples are available for replication when needed. Our results, especially the cytokine responses, have been confirmed by manual and unsupervised analysis. Furthermore, during assay optimization and validation, samples were run in duplicates.
Randomization	Samples were randomised prior to performing cytokine measurements in nasal and serum samples, as well as IgG serological tests in serum. Samples for flow cytometry were randomly collected from individuals that consented specifically for nasal scrape collection.
Blinding	All investigators were blinded to the categorization of the study participants (the hospitalized patients) until after producing all the data. The study groups were determined at data analysis based on the SARS-CoV-2 serology and RT-qPCR status.

Reporting for specific materials, systems and methods

We require information from authors about some types of materials, experimental systems and methods used in many studies. Here, indicate whether each material, system or method listed is relevant to your study. If you are not sure if a list item applies to your research, read the appropriate section before selecting a response.

Materials & experimental systems

n/a	Involved in the study	
[ ]	[x]	Antibodies
[x]	[ ]	Eukaryotic cell lines
[x]	[ ]	Palaeontology and archaeology
[x]	[ ]	Animals and other organisms
[ ]	[x]	Human research participants
[x]	[ ]	Clinical data
[x]	[ ]	Dual use research of concern

Methods

n/a	Involved in the study	
[x]	[ ]	ChIP-seq
[ ]	[x]	Flow cytometry
[x]	[ ]	MRI-based neuroimaging

Antibodies

Antibodies used	Nasal cell panel<br>CD45 Alexa Fluor 700 (Cat # 368514, Lot # B248834, Clone 2D1, Biolegend; 1:40)<br>CD3 APC (Cat # 300439, Lot # B278610, Clone UCHT1, Biolegend; 1:50)<br>CD66b PE (Cat # 12-0666-42, Lot # E18062-104, G10F5, eBiosciences; 1:80)<br>Anti-human IgG conjugated to horseradish peroxidase (Cat # ODL 150/10; Lot #103183, Omega diagnostics)
Validation	Same lot of fluorochrome-conjugated antibodies were used in all the flow cytometry experiments. All antibodies ware mouse-anti human and were titrated on appropriate human cells prior to use. All the flourochrome-conjugated antibodies are commercially available and are validated for flow cytometry applications by the vendor.<br>CD45 Alexa Fluor 700 - Nat Commun 2019 Feb 28;10(1):975. doi: 10.1038/s41467-018-08267-7.<br>CD3 APC - Nat Biotechnol. 2019 Mar;37(3):259-266. doi: 10.1038/s41587-019-0033-2.<br>CD66b PE - Oncoimmunology. 2018; 7(6): e1438108.

Human research participants

Policy information about studies involving human research participants

Population characteristics	All participants were over 18 years old. The demographic information is specified in Table 1 of the manuscript.
Recruitment	We prospectively recruited patients using the tier one sampling strategy from the International Severe Acute Respiratory and Emerging Infection Consortium (ISARIC) Clinical Characterisation Protocol (CCP). Briefly, patients over 18 years old were approached for informed written consent if they met inclusion criteria: severe acute respiratory infection (SARI) with suspected or confirmed SARS-CoV-2. Patients who did not demonstrate capacity at the time of approach were recruited using consultee assent, in line with the published protocol with consent confirmed retrospectively where possible after patient recovery. In addition, we recruited healthy community participants with no acute intercurrent or chronic illness as a healthy control group. All healthy participants, recruited immediately before (November 2019-March 2020) and after (September 2020-October 2020) the first peak in Malawi's reported COVID case count, were confirmed as seronegative for HIV-infection and had no known medical conditions.<br><br>Lack of critical care facilities precluded universal recruitment and sampling in the most severe cases, and our patient population may therefore not be entirely representative of all participants with SARS-CoV-2 induced SARI.
Ethics oversight	The project received ethical approval through the Malawi National Health Science Research Committee (NHSRC, 20/02/2518 and 19/08/2246) and Liverpool School of Tropical Medicine (study sponsor) Research Ethics Committee (LSTM REC, 20/026 and 19/017).<br><br>Approval for the SARS-CoV-2 ELISA verification samples was obtained from the College of Medicine Research Ethics Committee (COMREC, P.05/20/3045)

Note that full information on the approval of the study protocol must also be provided in the manuscript.

Flow Cytometry

Plots

Confirm that:

- [x] The axis labels state the marker and fluorochrome used (e.g. CD4-FITC).
- [x] The axis scales are clearly visible. Include numbers along axes only for bottom left plot of group (a 'group' is an analysis of identical markers).
- [x] All plots are contour plots with outliers or pseudocolor plots.
- [x] A numerical value for number of cells or percentage (with statistics) is provided.

Methodology

Sample preparation	Nasal cells were obtained using rhinoprobes (nasal scrapes) from each nostril and put into a tube containing 'RPMI and 10% fecal bovine serum (FBS)' [Complete media]. The cells were dislodged from the rhinoprobe and washed in complete media. Cells were then stained with a cocktail of antibodies before acquisition on an BD Fortessa flow cytometer (BD Biosciences).
Instrument	BD Fortessa (11 colour; 5-Blue 3-Red 3-Violet)
Software	Data collection: DIVA<br>Data analysis: FlowJo v10
Cell population abundance	No cell populations were sorted.
Gating strategy	We gated for singlets within the FSC-H and FSC-A plot, and then gated for CD45+ (leucocyte). From the CD45+ gate, we gated for CD66b+ (Neutrophils) or CD3+ (T cells) in the CD66b PE and CD3 APC flow plot.

- [x] Tick this box to confirm that a figure exemplifying the gating strategy is provided in the Supplementary Information.
